# Identification of models describing gene expression data leveraging machine learning methods

**DOI:** 10.1098/rsfs.2025.0014

**Published:** 2025-08-22

**Authors:** Lucas F. Jansen Klomp, Elena Queirolo, Janine N. Post, Hil G. E. Meijer, Christoph Brune

**Affiliations:** ^1^ Mathematics of Imaging & AI, Department of Applied Mathematics, University of Twente, Enschede, The Netherlands; ^2^ Developmental BioEngineering, University of Twente, Enschede, The Netherlands; ^3^ IRMAR, University of Rennes, Rennes, France

**Keywords:** ODE modelling, graph neural network, gene regulatory network, scRNA-seq

## Abstract

Mechanistic ordinary differential equation models of gene regulatory networks are a valuable tool for understanding biological processes that occur inside a cell, and they allow for the formulation of novel hypotheses on the mechanisms underlying these processes. Although data-driven methods for inferring these mechanistic models are becoming more prevalent, it is often unclear how recent advances in machine learning can be used effectively without jeopardi zing the interpretability of the resulting models. In this work, we present a framework to leverage neural networks for the identification of data-driven models for time-dependent intracellular processes, such as cell differentiation. In particular, we use a graph autoencoder model to suggest novel connections in a gene regulatory network. We show how the improvement of the graph suggested using this neural network leads to the generation of hypotheses on the dynamics of the resulting identified dynamical system.

## 1. Introduction

With the advent of single-cell sequencing technologies that quantify gene expression, computational methods have become paramount in understanding intracellular biological processes [ 1,2]. Gene regulatory networks (GRNs), for example, describe the connections between downstream transcription factors and target genes [3 ]. Such networks are represented as directed graphs, indicating the relations between different genes in the network. Many methods exist to infer GRNs from gene expression data, and while these methods are often well suited to tasks such as clustering and identifying key nodes, individual functional relations between nodes in the network are typically not well represented [4–7 ]. This lack of reliable representation limits the use of data-driven inferred GRNs for dynamical modelling of intracellular processes, where such functional relations are needed to obtain accurate predictions. For example, when the model parameters are perturbed, different GRNs can result in remarkably different behaviour. In this work, we ask how we can leverage successes in the machine learning field to identify more informative mechanistic models describing intracellular processes based on single-cell RNA-sequencing data (scRNA-seq).

Nowadays, scRNA-seq data are commonly used in computational methods, and consist of the gene expression of many (approx. 500−10 000) cells harvested from the same population [ 1,2 ]. Hence, the data consist of an n×m matrix, where n is the number of measured genes and m is the number of cells. Sometimes, time points can also be associated with individual cells, either because cells were harvested at specific time points during the experiment or through computational methods inferring pseudotime trajectories [8,9 ]. The high resolution of scRNA-seq allows for the application of more advanced techniques that cannot be used on bulk data. In particular, the large number of samples makes machine learning a promising avenue to explore and obtain meaningful insights from single-cell data.

One class of machine learning methods used to study scRNA-seq data is tasked with recovering the underlying GRN based on (in principle) data collected from one population of cells. These methods often rely on an encoding –decoding scheme to predict links between nodes in the GRN. Examples include methods like DeepSEM [10 ], which uses structural equation modelling in its encoder and decoder schemes, and GNNLink [11 ], DeepRIG [12 ] and DGCGRN [13 ], which leverage the inherent graph structure underlying the data by using a graph autoencoder (GAE) to learn node embeddings and subsequently predict links between genes. Another recent method, GENELink, uses the graph attention mechanism to recover links between nodes [14 ]. In general, deep learning-based GRN inference methods report much higher accuracy than classical statistics-based methods, although good interpretability of the resulting networks is rarely achieved. To remedy this, we explore how we can fit a biologically interpretable model using the output of a graph autoencoder.

Specifically, we focus on methods that retrieve a dynamical model based on data. In the literature, the focus is sometimes on recovering the dynamics underlying a temporal process captured in the single-cell data as its own goal, whereas in other cases, the dynamics are instrumental in obtaining a GRN. Some notable references in the field include the method SCODE, which aims to reconstruct the GRN based on inferred parameters of a linear ordinary differential equation (ODE) model [15 ]. In RNAForecaster, a neural ODE (nODE), an ODE where a neural network represents the right-hand side, is used to recover gene expression dynamics [16 ]. Furthermore, PHOENIX [17 ] aims to build a more biologically informed model and fits a model based on Hill functions to the data while using a linear ODE to regularize model training.

From a practical perspective, the inferred computational models resulting from these machine learning methods can be used to identify key genes influencing the biological process. Perhaps more importantly, the models can be used to obtain viable hypotheses on what would happen if particular genes are knocked down or if connections are modified. In this second use case, having a precise view of which functional relations exist between genes is crucial.

It is natural to encode a GRN as a directed graph, in line with a variety of other works such as [13,18 ], where each vertex $x_{i}$ represents the expression of a given gene and each edge $x_{i}\to x_{j}$ indicates that the expression of a target gene $x_{j}$ depends on the expression of the source gene $x_{i}$. We then denote with V the set of vertices of the graph, that is, $V=\left\{x_{1},x_{2},\ldots ,x_{K}\right\}$ and E the set of edges. A directed graph can also be represented as an adjacency matrix $A\in [0,1{]}^{K\times K}$, where we interpret $A_{ij}$ to be the probability of the existence of the edge $x_{i}\to x_{j}$. Based on the given GRN, we can construct a model of the form

(1.1) $$\tau \dot{x}=-x+H(x,W_{\text{in}},u).$$

Our challenge is to find the optimal parameters $p=\left(W_{\text{in}},u,\tau \right)$, where the sense of this optimality will be specified later in § 2.2 . Here, σ is an activation function. This formulation resembles commonly used models in biochemical applications, where Hill functions are often used to describe the nonlinearity σ. We say that the parameters p are compatible with a graph given by the adjacency matrix A if the matrix $W_{\text{in},ij}$ is non-zero if and only if $A_{ij}$ is non-zero for all i,j. An advantage of this parameter formulation is that the parameter size is completely determined by the phase space dimension, that is, the number of modelled genes, rather than the specific structure of the GRN. This also permits us to compare all parameters associated with a given time series, even when they are not compatible with the same graph.

**Figure 1.**
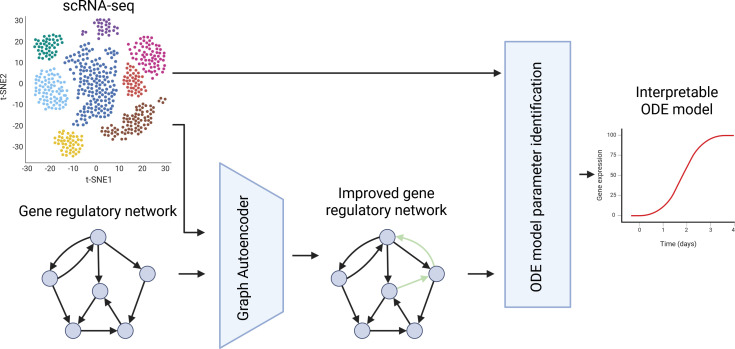
Overview of our proposed framework. We use scRNA-seq data to adjust an available GRN using a graph autoencoder model. Subsequently, we use the adjusted graph topology as a basis for an ODE model describing the scRNA-seq. By fitting the parameters in this ODE model, we obtain an interpretable ODE model that can be used to describe the data and to predict what happens when chosen parameters are adjusted.

In this article, we present a framework that combines machine learning methods to infer a model of the form (1.1) (an overview of the framework is shown in figure 1). In particular, we use a graph autoencoder to update and improve a GRN obtained from prior knowledge or other GRN inference methods. Based on the improved GRN, we fit the parameters of a mechanistic nonlinear ODE model (1.1) to the available data.

Our framework highlights how recent successes in the deep learning field can be leveraged in the construction of mechanistic ODE models. We evaluate the predictive properties of the resulting ODE models, including their suitability for extensive parameter sensitivity analysis through a bifurcation analysis or even more rigorous identification of parameter regimes around the inferred model parameters [18 ].

## 2. Methods

In this section, we first introduce a GAE to improve GRNs based on scRNA-seq data. Subsequently, we describe how the output of this GAE is used to infer parameters of a nonlinear ODE model. Finally, we introduce benchmarking data to test the performance of our framework.

When considering a GRN, the dynamics are strictly related to the graph that supports it. In many cases, such as models with ramp functions as nonlinearities, it is possible to construct a characterization of the supported dynamical behaviour across the entire parameter space ( see, for example, the DSGRN framework [18 ]) or explore parameter regions using a Boolean network view [19 ]. Recent results indicate that such results also apply to a much broader class of nonlinearities [20,21 ]. While the graph structure is known to be critical in the definition of the dynamics, it is also well known that the effects of the graph structure on dynamics are far from being fully understood. For this reason, we first concentrate on the task of improving known graphs using a GAE before fitting parameters of an ODE model.

### 2.1. Graph autoencoder to predict missing edges in a gene regulatory network

Assuming that an initial (imperfect) graph is known, we want to improve its accuracy based on available scRNA-seq data. We assume that a graph is given where one or a few edges that are in the true graph are obscured. Specifically, a prior knowledge graph $G_{\text{PK}}=\left(V,E\right)$ and the associated adjacency matrix A are given as input imbued with node features $X\in {\mathbb{R}}^{|V|\times N}$ , where N is the number of cells included in the scRNA-seq dataset and |V| is the number of nodes in the GRN. In our case, we assume X includes gene expression data measured in many cells, and is stored in pairs that contain cells along with associated time points per cell, that is, we also have access to $t\in {\mathbb{R}}^{N}$ that includes these time points.

In this section, we use $G_{\text{PK}}$ and X as inputs for a GAE , with the explicit goal of learning node embeddings. These node embeddings are vectors that, in a well-trained network, store representative features of a particular node. Based on these node embeddings, we can decode the network by computing a similarity measure between the node embeddings of different nodes in the network. Such a scheme has previously been used successfully to infer GRNs [12 ].

We then encode the node features to a latent embedding using a 2-layer neural network, where each layer is given by a GraphSAGE layer [22 ]. Here, $X_{i}$ contains the ith row of X, that is, all measurements for a single node i, and ReLU is the rectified linear unit activation function:


$$\left\{ \begin{array}{l} X_{i}^{0}=X_{i},\\ X_{i}^{n+1}=\text{ReLU}(W_{1}X_{i}^{n}+W_{2}\cdot {\text{mean}}_{j\in N(i)}X_{j}^{n}). \end{array}\right.$$

Here, N(i) denotes the neighbourhood of node i, defined as all nodes that are at most one edge away in the graph (each edge is assumed to be undirected for the purpose of propagating signals through the GraphSAGE layer). The weights $W_{1}$ and $W_{2}$ are learnable matrices. Effectively, this neural network layer aims to aggregate information from neighbouring nodes to obtain a representative encoding of the features of nodes in the network. After a latent representation Z of the node features is obtained using the encoder, an adjusted adjacency matrix for the graph is obtained by decoding the representations:


$${\hat{A}}_{ij}=(\sigma (Z^{T}W_{3}Z){)}_{ij}$$

with


$$\sigma (x)=\frac{1}{1+{\text{e}}^{-x}}.$$

Here, the goal is to assign to each predicted edge a score in the interval [0, 1] , where 0 indicates the certitude of no connection being present and 1 indicates the certitude of a connection being present. The sigmoid function σ maps the output from the GAE to this interval [0,1] and is often used for this purpose in machine learning applications. Again, the matrix $W_{3}$ contains learnable parameters, and ${\hat{A}}_{ij}$ denotes the estimated entry in the output adjacency matrix $\hat{A}$. This decoding scheme is also used in DeepRIG [12 ].

For training the network, we use Binary Cross Entropy (BCE) loss to optimize the weights $W_{1},W_{2},W_{3}$ of the network, where $E_{T}$ denotes the set of true edges and non-existent edges the network is trained over, and $A_{ij}\in \left\{0,1\right\}$ denotes the label of edge $(i,j)\in E_{T}$ ($A_{ij}=1$ if (i,j)∈E and $A_{ij}=0$ if (i,j)∉E):


$$L_{\text{GAE}}=\frac{1}{|E_{T}|}\sum_{(i,j)\in E_{T}}A_{ij}\log({\hat{A}}_{ij})+(1-A_{ij})\log\left(1-{\hat{A}}_{ij}\right).$$

**Figure 2.**
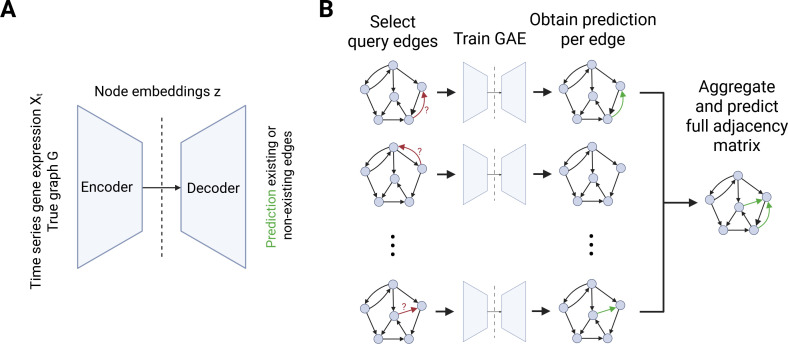
Graph autoencoder for predicting additional connections in GRNs. ( A) GAE network architecture. The encoder consists of a two-layer graphSAGE network. The decoder consists of an inner-product decoder with learnable weights. ( B) Procedure for improving the GRN. The GAE is trained for each edge for which a prediction is desired, and the results are subsequently combined to obtain an improved graph structure.

In the practical use case for the GAE model, the GAE is trained on an available prior knowledge GRN along with available scRNA-seq data. Through an iterative approach training the encoder –decoder scheme for each possible edge that could be added to the graph, we obtain the connections in the graph that are most likely to exist given the input data (figure 2B). We can then use the inferred corrected network as an improved graph structure for downstream applications. This leads to an improved network $G_{I}=\left(V,E\right)$ characterized by the adjacency matrix $\hat{A}$. We note that the GAE is time-invariant, and does not require pseudotime trajectories to adjust the GRN. The GAE presented here differs from the graph autoencoder-based GRN inference method DeepRIG [12 ] by using a different GNN layer in the encoding step, by relying on a general prior knowledge network rather than a GRN inferred using WGCNA, and by introducing a leave-one-out policy to infer connections.

Our GAE architecture consists of two GraphSAGE layers, each with a hidden dimension 200. Hence, the dimension of the learned node embeddings is also 200. During training, we dropout edges with a probability $p_{e}=0.2$ and apply a dropout layer to the output of the first GraphSAGE layer with a probability $p_{d}=0.3$ to prevent the model from overfitting the known edges. Such dropout layers are particularly important since we often consider small networks, where the risk of overfitting is large.

### 2.2. Model parameter inference

Based on the optimized GRN structure, we construct mechanistic models that describe temporal intracellular processes. In our case, we fit the already introduced nonlinear ODE model to the experimental data:

(2.1) $$\tau \dot{x}=-x+H(x,W_{\text{in}},u),$$

where we choose the nonlinearity to be


$$\begin{array}{c} H_{i}(x,W_{\text{in}},u)=\frac{u_{i}+\sum_{W_{\text{in},ji}>0}W_{\text{in},ji}x_{j}^{3}}{1+u_{i}+\sum_{W_{\text{in},ji}>0}W_{\text{in},ji}x_{j}^{3}+\sum_{W_{\text{in},ji}<0}-W_{\text{in},ji}x_{j}^{3}}. \end{array}$$

Such a Hill function-based model is commonly used for modelling intracellular signalling [23,24 ]. Here, $x\in {\mathbb{R}}^{N}$, τ∈ℝ, $u\in {\mathbb{R}}^{N}$ and $W_{\text{in}}\in {\mathbb{R}}^{N\times N}$. We note that fitting parameters for this network can be seen as training a one-layer graph neural ODE, where this single neural network layer is given by the right-hand side of the ODE. We remark that the weights in this model are not related to the ones of the GAE introduced in § 2 .1.

We assume that data are provided in pairs of time point and gene expression of genes at that time point. With $(t_{i},X_{i})=D_{i,t}\in D$ we denote the measured expression of gene i at time point $t_{i}$. We can use the comparison between the model simulation at time $t_{i}$, $x_{i}(t_{i})$, and the data $D_{i,t}$ to evaluate the performance of the fitted dynamical model.

Specifically, we improve the fit of the model to the given experimental data by minimizing the mean squared error between data and model simulations:


$$\begin{array}{c} L_{\text{model}}=\sum_{D_{i,t}\in D}|D_{i,t}-x_{i}(t){|}^{2}. \end{array}$$

Moreover, we aim to have a minimal model describing the data, and therefore we add an *L*1 loss term applied to the parameters. This L1 regularization term strikes a balance between pushing parameters towards zero against their importance in recovering the correct time-series data, and is commonly used as a way to promote sparse solutions in optimization problems [25 ]. This is represented as


$$\begin{array}{c} L_{\text{p}}=\sum_{p_{i}\in p}|p_{i}|, \end{array}$$

with $p=\left(W_{\text{in}},u,\tau \right)$.

**Figure 3.**
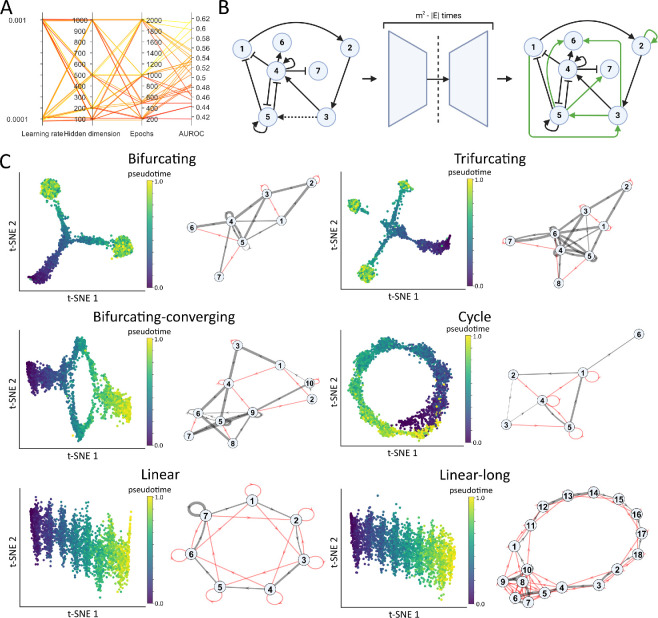
(A) Results from hyperparameter tuning for our graph autoencoder model introduced in § 2.1 . Each parallel axis denotes a hyperparameter choice, and the colour of the line indicates the AUROC for the mCAD network with 2000 simulated cells as provided in BEELINE [3 ]. The configuration of learning rate 0.0001, hidden dimension 200 and 1000 epochs performed best. ( B) Application of the GAE on the bifurcating network. *L* *eft *. The damaged bifurcating graph given as input, where the dashed line indicates the edge removed from the original true graph. *R* *ight *. The improved graph structure based on the output of the GAE. Edges that were added are indicated in green (threshold score for adding the edge was set to 0.9). *C* *ent* *re* . A representation of the GAE. The training procedure is repeated $m^{2}-\left|E\right|$ times to build the adjusted output graph, where m is the number of nodes and |E| is the number of edges. ( C) Results for six representative networks based on our graph autoencoder network. For each network, we show a dimension reduction using t-SNE for the data simulated using BoolODE, where each dot indicates one sample in the dataset. Here, blue indicates t=0 and yellow indicates t=1 (the end point of the simulated data). Next to each data visualization, we show statistics for networks improved using our GAE network. The grey edges indicate edges that are in the true network, and the thickness of the edge indicates how well this edge is recovered using the GAE (rank among all inferred connections/amount of possible connections in the graph). The red lines indicate edges that are often inferred using the GAE network (mean score >0.5) that are *not***** in the true underlying network.

Finally, in some cases, the trajectories in the data split up from a common starting point (see, for example, the pseudotime analysis shown in figure 3C). In these cases, we add the initial conditions for each splitting trajectory as tunable parameters, and add a term to the loss function ensuring that these initial conditions are as close as possible to the starting point in the reference data. In this way, we recognize that measured initial conditions can be noisy:


$$\begin{array}{c} L_{\text{IC}}=\max_{i}|D_{i,0}-x_{i}(0)|. \end{array}$$

A similar regularization on the initial conditions has been used in the context of physics-informed neural networks [26 ]. Combining terms, the complete loss function becomes


$$\begin{array}{c} L=L_{\text{model}}+\lambda_{\text{p}}L_{\text{p}}+\lambda_{\text{IC}}L_{\text{IC}}. \end{array}$$

If multiple trajectories are available, the losses for each individual trajectory are summed. Here, $\lambda_{\text{IC}}$ and $\lambda_{\text{p}}$ are hyperparameters used to scale the importance of the different terms in the loss function. In our experiments, we set $\lambda_{\text{p}}=0.001$ and $\lambda_{\text{IC}}=10$. The purpose of these hyperparameter settings is to introduce a hierarchy of importance for different terms in the loss function. The fit to the time-series data along with initial conditions close to the starting point in the data are prioritized. Only after a reasonable fit for these terms is achieved, excess connections and parameters are slowly adjusted to zero if they do not contribute meaningfully to the fit.

There are many methods for optimizing parameters for a given system of ODEs. Although many approaches achieve similar results, we use a global optimizer of differential evolution type, adaptive DE/rand/1/bin [27 ]. Let us remark here that determining the correct graph in the previous step is fundamental to decrease the size of the parameter space. If a complete graph was considered, we would need to fit a full matrix $W_{\text{in}}$, consisting of $n^{2}$ parameters. Here, instead, we fit only the necessary non-zero parameters, so that $W_{\text{in}}$ has only |E| non-zero elements, where E denotes the set of edges associated with the improved graph $G_{I}$. We recognize that our model formulation is less flexible than other possible formulations. However, we emphasize that the smaller parameter space in our formulation pushes the model to use the available network structure, rather than relying on per-node flexibility to model time- series data.

### 2.3. Training data

To test our methods, we apply the GAE and model fitting to synthetic datasets generated using BoolODE [3 ]. Based on this method, six representative small GRNs are used as input to our method. We use the bifurcating, bifurcating-converging, trifurcating, cycle, linear and long linear graphs provided in this dataset ; see figure 3. These networks are well documented and have been used to test and benchmark GRN inference methods [3,12 ].

Next to the graph structures provided for these six graphs, synthetic scRNA-seq data are generated. For each of the graphs, we have simulated 3000 cells using BoolODE [3 ] with maximum time 8. One time point is randomly selected for each of these 3000 simulations, and the gene expression at this time point is added to the synthetic scRNA-seq dataset. Hence, our synthetic dataset consists of the gene expression of 3000 cells along with an associated time point for each cell. While a noisy dynamical system is used to simulate the gene expression over time, we note that in our current setup, we do not randomly drop out gene expression for specific genes. To fit ODE models to th ese data, we collect all cells within bins of pseudotime with width 0.1 to get time-series data to fit the ODE model. Afterwards, we linearly scale the time-series data to the interval [0.1, 0.9] to obtain data that can be represented using equation (2.1). In case multiple clusters or trajectories are available, we construct time series in this way individually for each cluster or trajectory. In these cases, we scale the time series based on all available time series, in order to preserve differences in expression between the available data.

In addition to synthetic data, we evaluate our GAE on one large real-world GRN describing human embryonic stem cell (hESC) differentiation using scRNA-seq data as described by Chu *et al.* [28 ]. We use the pre-processed GRN provided by Pratapa *et al.* [3 ] , which consists of putative connections between 130 transcription factors (TFs) and 18 104 target genes based on ChIP-seq data . While the full network can be used, the degree distribution of this network is heavily skewed with the 130 TFs being the only nodes with outgoing connections. For this reason, we have chosen to consider only a subnetwork of this large network, which entails the 130 TFs and connections between these TFs. This real-world GRN is still much bigger than the small synthetic networks we consider. Single-cell RNA-seq data are available for 758 cells, and the considered TF network has 130 nodes and 3866 edges. In § 3.1 , we use this real-world network to assess the scalability of our graph autoencoder approach to improving GRNs.

For each of the six representative networks, we first consider the original graph ( *true* *graph* ), and then remove one connection from the true graph topology to obtain the *damaged graph* . We then use the damaged graph as input for our GAE, which leads to an *improved graph* . We compare the features of the original BoolODE model with two dynamic models obtained based on the damaged and improved graphs. First, we fit the parameters of the same model based on the damaged graph, and then we fit the parameters of the model based on the improved graph. The purpose of this comparison is to evaluate how the additional GAE step influences key properties of the resulting dynamic model.

## 3. Results

We now focus on the results of the two key parts of our proposed framework. First, we show the results of the GAE when used to recover edges in a GRN. Next, we discuss the results of fitting an ODE model based on the graphs.

### 3.1. Graph autoencoder network recovers unseen connections in gene regulatory networks

In this section, we focus on the performance of our GAE described in § 2.1 with the purpose of improving the graph structure of GRNs.

Before testing our GAE on various GRNs, we have tuned the hyperparameters for the GAE model. We have used the mCAD network with synthetic scRNA-seq data provided through BEELINE [3 ] as a validation network to test the effect of key hyperparameter choices. The results of this parameter tuning are shown in figure 3A. Here, we see that a learning rate of 0.0001, a hidden dimension of the encoder of 200 or 500 and 1000 or 2000 training epochs perform well for the mCAD network. Since a learning rate of 0.0001, a hidden dimension of 200 and 1000 training epochs performed best for the validation network, we use these parameter choices for the remainder of this section.

First, we show the application of our GAE network in a typical use case (figure 3B). For this example, we take the bifurcating graph as provided in the BoolODE dataset and remove one edge from this graph [3 ]. We use the damaged graph along with available scRNA-seq data as input for our GAE, which we train once for each connection that could be added to the graph, predicting whether those connections exist. Each of these training runs leads to a score associated with the predicted edge between 0 and 1. Combining the results from the different training runs results in a new graph structure, where new edges are added to the graph. In this example case, we recover the removed edge using a score threshold of 0.9, but we also predict some additional edges to be in the graph that were not in the true graph.

**Figure 4.**
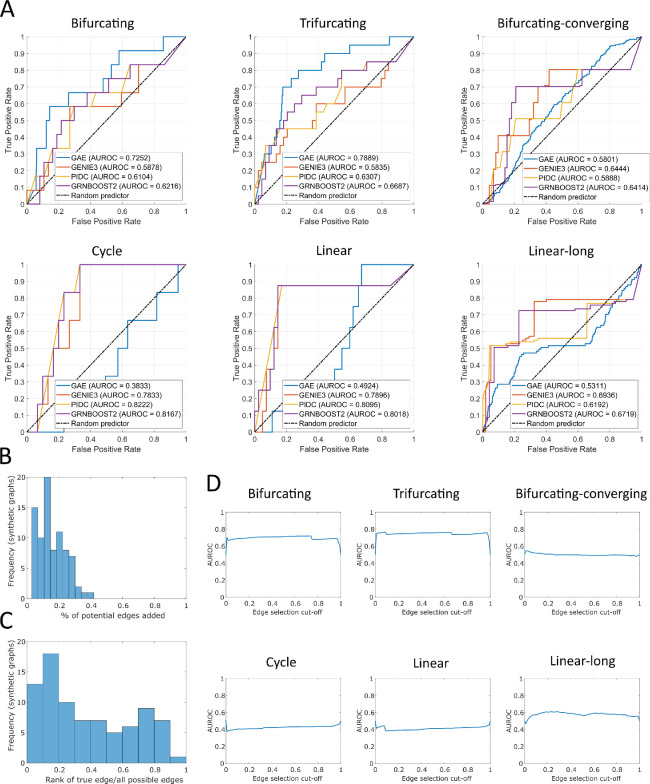
Statistics for the performance of the GAE. ( A) ROC curves for each of the tested synthetic GRNs (figure 3C), comparing the performance of our graph autoencoders against the methods GENIE3, PIDC and GRNBOOST2 [6,29,30 ] as implemented in BEELINE [3 ]. A higher area under the ROC curve (AUROC) corresponds to a better binary classifier. ( B) The total amount of edges added to the graph when using the GAE to recover a removed edge (threshold for inclusion is set to a score of 0.9) divided by the number of potential edges that can be added. This is an indication of how many spurious connections are expected to be added to the graph if the GAE is used to improve a GRN. ( C) Rank of the edge removed from the network compared with all possible edges in the graph. ( D) Effects of changing the decision threshold for adding connections on the AUROC.

To gain insight into the performance of our GAE on multiple graphs, we apply our GAE for each edge in each of the six representative graphs described in § 2.3 (figure 3). We have subsequently computed ROC curves for each of these networks to evaluate the performance of our GAE as a binary classifier that predicts additional edges in the GRN (figure 4A). Here, we compare the performance of our proposed graph autoencoder with three alternative network inference methods, GENIE3, GRNBOOST2 and PIDC [6,29,30 ], as implemented in BEELINE [3 ]. We see that our GAE outperforms these three GRN inference methods on the bifurcating network and the trifurcating network. Performance in the bifurcating-converging network is similar to that in the compared methods. However, we also see that the performance of our GAE is similar to that of a random predictor for the cycle, linear and long linear synthetic networks.

We also present some summary statistics for the performance of the GAE on the six representative graphs in figure 4. We see that the edges removed are typically recovered earlier than other spurious edges in the graph (figure 4C). Moreover, we look into how many edges we add to the graph if we include all edges given a score higher than 0.9. We find that a pproximately 15 % of all possible edges are added (figure 4B). Hence, our autoencoder significantly increases the amount of connections for the tested GRNs, even if only one true edge is removed. We also consider how the score threshold used to determine if an edge is added affects the performance of our method (figure 4D). We see that, in general, the AUROC does not change much after tuning this decision threshold, indicating that most edges get a score that is very close to 0 or very close to 1.

To gain more insight into where our GAE performs well and where it underperforms, we show two key metrics for each of the synthetic graphs (figure 3C). First, we show how well each of the removed edges is recovered if it is removed from the graph. This is indicated by the thickness of the grey edges in the graphs shown (all grey edges are edges in the true graph). Specifically, we calculate the rank of the removed edge among all possible edges in the graph R(i,j) *,* based on the output score for each edge from the GAE. For a graph with m nodes, the edge weight based on this rank is linearly related to


$$\frac{m^{2}-|E_{I}|-R(i,j)}{m^{2}-|E_{I}|}.$$

Here, $E_{I}$ denotes the set of edges of the damaged graph used as input for the GAE. If the removed edge is easily recovered, this defines a larger edge weight.

Second, we indicate in red edges that are often recovered based on the output of our GAE that are not in the true graph. To identify which erroneous edges are often recovered, we take the mean of the output score of the GAE trained for each of the edges in our network, and say that an edge is often recovered if this mean score is larger than 0.5.

We see that the graph structure has a large influence on how well the GAE recovers removed edges. For example, many edges in the bifurcating graph are recovered correctly, whereas the linear network is difficult to encode and decode using the GAE. This difficulty may be caused by the fact that our two-layer GraphSAGE-based encoder can only use information from up to two edges away in the damaged graph to obtain a node embedding for all nodes in the system. Moreover, we typically get better results when recovering self-loops or edges that are part of a direct feedback loop (i.e. even if the edge is removed, there is an edge going the other direction). This makes sense based on our GAE architecture, since information is still shared between the two nodes involved in the edge in these cases.

There are also some patterns visible when considering the edges that are often erroneously added to the graphs. First, there is a bias towards self-loops. This is again due to the way that the GraphSAGE layer is set up, in which the nodes always pass information to themselves. Next, there are some cases where ‘indirect ’ connections (edges between nodes that are at a distance of 2 in the true graph) are erroneously found. Likely, the scRNA-seq data for these connected nodes are similar, which, along with similar information being passed to these nodes through the GAE (since the source nodes are connected), leads to similar node embeddings for both source nodes. In turn, this makes it difficult to discern which node truly has a connection to a downstream node. Similarly, we see a clear bias in the linear networks towards adding connections that skip one node in the chain. Potential improvements could be made to the performance of the GAE by adapting the architecture of the GAE so that global features can be taken into account, e.g. through positional encoding.

To see how our approach generalizes to larger real-world networks where data are more noisy, we have tested our GAE using the scRNA-seq data available for hESCs along with a cell-type specific network based on ChIP-seq data as provided in BEELINE [3,28 ]. We consider a transcription factor network of 130 nodes and 3866 edges. Since we now have many edges available, we can use the standard training procedure for graph autoencoders, where we split off test and validation edges from the network before training and use these edges to assess the performance of the network. When using the full transcription factor network, we obtain an average AUROC on the set of test edges of approximately 0.84 over 20 runs. This shows that our GAE is capable of predicting unseen connections based on noisy experimental data if many good examples of edges are available.

**Figure 5.**
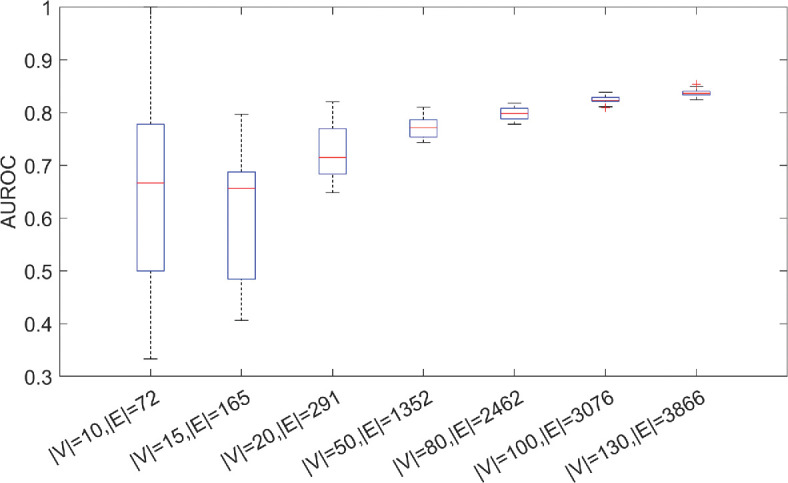
Performance of our GAE on a real-world GRN and experimental data describing hESC differentiation as obtained from BEELINE [3,28 ]. We show the AUROC over 10 training runs for subnetworks of different sizes built based on the ChIP-seq network, constrained to only transcription factors. Each subnetwork is constructed by taking the top N nodes based on degree in the ChIP-seq network and all connections between these top N nodes.

We subsequently ask how many nodes and edges are needed in the TF network to obtain a good prediction of unseen edges. To investigate this, we have created subnetworks of the top N nodes with the highest degree based on the TF network, and we have trained our GAE using these subnetworks. We show that our GAE is better on average than a random predictor even for a small networks of 10 nodes and 72 edges. Importantly, performance and stability increase as more examples of edges are available (figure 5). Whereas overfitting must be explicitly avoided in the small synthetic networks, this shows the possibility of avoiding the leave-one-out strategy used for the small synthetic graphs if more data are available.

### 3.2. Dynamical models based on improved networks

Based on the improved network topology, we fit an ODE model to trajectories in the synthetic scRNA-seq data generated using BoolODE for the true graph structure (see § 2.3 for details). In this section, we focus only on the bifurcating network. Specifically, we fit an ODE model to the two trajectories in the bifurcating network (as seen in figure 3). We follow the example shown in § 3.1, where the edge from node 3 to 5 is removed from the graph. Hence, we consider the two distinct cases for optimized ODE models introduced in § 2.3 that are based on the following graphs:

— The damaged bifurcating graph with the edge from node 3 to 5 removed.
— The improved bifurcating graph using our GAE after the edge from node 3 to 5 was removed.

**Figure 6.**
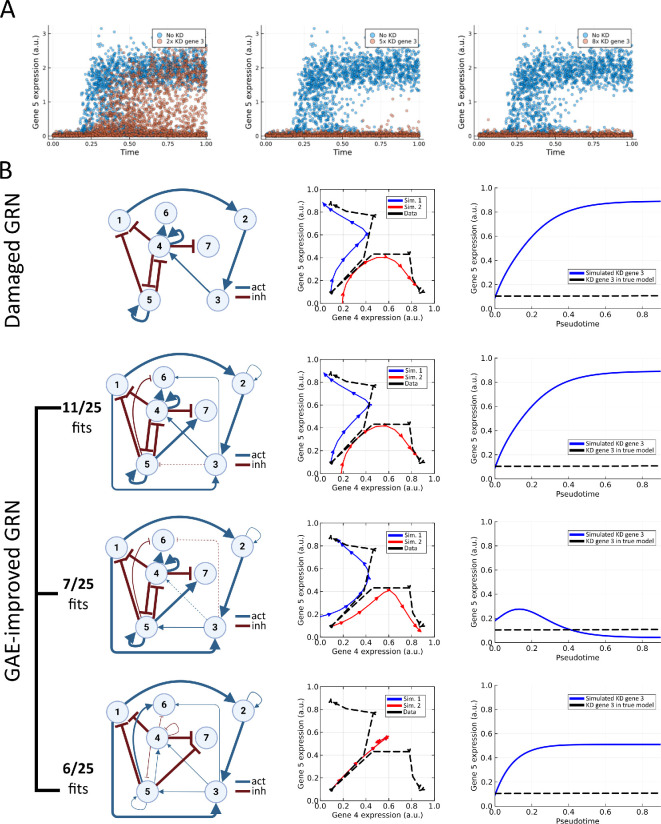
(A) Results from simulated knockdown experiments of gene 3 in BoolODE for the true bifurcating graph. We show that knockdown of gene 3 should result in lower expression of gene 5 (red) compared with a model without knockdown (blue). ( B) Results from fitting the ODE system to the damaged bifurcating graph (removing the edge from node 3 to node 5) and the improved bifurcating graph based on the graph autoencoder. For each graph, we show three results. First, we show all commonly found parameter settings after the fit ( *left* ). Then, we show a phase portrait for genes 4 and 5 with simulations of the fitted model (* middle *). These simulations should follow the splitting trajectories found in the data (black, dashed line in this figure, synthetic input data are shown in figure 3C). Finally, we show the result of knocking down gene 3 on a simulation of the expression of gene 5 (* right *). This simulated knockdown experiment should correspond to the simulated knockdown experiment in (A) .

Considering these two networks and their associated ODE model, we find parameters for each model to fit the synthetic data for the true bifurcating network simulated using BoolODE (figure 6A). After testing the descriptive performance of the fitted models by comparing model simulations to the synthetic data, we test the performance of the fitted models in predictive tasks. To do this, we perform an *in silico* knockdown experiment. In particular, to knock down a chosen gene $x_{i}$ in the network, we modify ${\dot{x}}_{i}$ to be


$$\begin{array}{c} \tau {\dot{x}}_{i}=-(k+1)x_{i}+H{\left(x,W_{\text{in}},u\right)}_{i}, \end{array}$$

where k denotes the strength of the knockdown, which we set to k=2. In this way, we evaluate whether the parameters fitted for the alternative graph structures lead to meaningfully different results if the model is used to predict the outcome of an experiment. This knockdown can also be represented in the BoolODE model used to generate synthetic data, leading to synthetic knockdown data to which the simulations of the fitted model can be compared (figure 6A). In BoolODE, we have modified the degradation rate to be 2 times, 5 times and 8 times the original degradation rate to simulate knockdown experiments, and compare our model simulations with the five times knockdown experiment.

After running our parameter fit 25 individual times for both graphs, we find that the desired bistable behaviour is always fitted correctly for the damaged GRN (figure 6B). However, once knockdown is simulated for gene 3 in this network, we observe an effect opposite to the desired effect as gene 5 expression increases. Fitting parameters for the GAE-improved network, we instead find that there are three alternative parameter choices resulting from our parameter optimization (figure 6B). In some fits, we find a network configuration that is very similar to the parameters found for the damaged GRN, in which case the *in silico* knockdown experiment gives a similar undesired result. However, in 7 of the 25 fits run, the added edge from gene 3 to gene 5 is given a larger connection strength, and we find knockdown simulations that are in line with the simulated true model. Finally, there are some cases where the system gets stuck in a local minimum where no bistability is present, in which case the characteristic negative feedback loop between nodes 4 and 5 is not recovered through the parameter fit. The final class of parameter fits is not shown since it only appeared once in the 25 fits run. This final parameter configuration does have the negative feedback loop between genes 4 and 5, but both nodes do not receive input from node 3. Instead, the model relies on the self-activation u to reconstruct the time-series data.

A key observation is that the models fitted for both configurations are capable of fitting the single-cell data for the two trajectories well, as exemplified by the simulations for gene 5, where a bump in expression is recovered for all the graphs considered (figure 6). Hence, even if key edges are removed from the graph, the model definition has enough flexibility to capture key dynamical features of the data. An exception to this is when the only incoming edge of a node is removed. In this case, the dynamics of the node must follow a linear system, typically leading to suboptimal solutions of the fitted ODE model. This result strongly suggests that fitting data alone is not an indication that a fitted model is correct and more rigorous analysis is necessary to evaluate the performance of a data-driven model.

To look at the graph structure resulting from the fit, we visualize $W_{\text{in}}$ to get a view of which connections are stronger and which connections are weaker in each of the fitted ODE models (figure 6B). By looking at these graphs, it is apparent that a choice is made for parameter fits for the model based on the GAE-improved graph: gene 3 can influence the negative feedback loop between genes 4 and 5 either through activating node 4 or through activating node 5. How these two effects are balanced subsequently determines the outcome of knockdown experiments. As this choice is not available for the damaged network, the models fitted for the damaged networks are consistent, but always incorrect.

Our results suggest that it is necessary to look at an ensemble of fits, rather than an individual fit, in order to obtain well-founded hypotheses on the underlying dynamical system. Given a reasonable formulation of the ODE model, there is too much freedom in selecting viable parameters when fitting to a single time-series data, so that many configurations of the graph lead to reasonable results of the dynamic model. Within this context, the GAE resented in this work is capable of suggesting new connections to add to the GRN. This helps to bridge the gap between the underdefined damaged network, where there is no possibility of recovering the correct mechanism, and the updated network, where finding the correct mechanism is not certain but is possible.

## 4. Discussion

In this work, we have explored how recent developments in machine learning can be used to aid in constructing interpretable mechanistic models for intracellular processes. In particular, we have constructed a GAE to suggest new connections in GRNs. Subsequently, we have fitted nonlinear ODE models to three graphs: the original true graph structure, a damaged graph structure where a key edge is removed, and an improved graph structure, the output of our GAE using this damaged graph as input. We have tested the results according to their ability to fit the dynamics of not only the true system, but also the one expressed by *in silico* knockdown experiments. Our work shows an example of how methods from deep learning have a place within mechanistic modelling, providing the interpretability of models that is often desired.

Starting from a given graph structure of the GRN and scRNA-seq data, our GAE can suggest additional edges to add to the small input graph. We have shown that this method works best on densely connected graphs, and performs worse on weakly connected graphs, such as simple rings. We present a training procedure that provides predicted edges even if only a very small GRN is available, and we show the scalability of our approach to real-world cases if a larger baseline GRN is available. In the architecture presented, we also show that there are areas of improvement for the constructed GAE, particularly a bias towards self-loops and edges to nodes that are two edges away in the true graph. Both of these fallbacks can be attributed to the structure of the two-layer GraphSAGE neural network used for encoding node features. They could be overcome by explicitly learning on global features of the graph rather than local features.

The fitting of an ODE system to the synthetic scRNA-seq data has demonstrated that even with a correct graph structure, there are a variety of parameters possible that lead to a simulation matching the data. This result, while not ideal, is compatible with the results from DSGRN [18 ] indicating that many parameter regions can support identical dynamical signatures. This shows that having the right graph structure is necessary but not sufficient for finding a correct model. This conclusion challenges the standards used in modelling applications, where a good fit to the experimental data is often considered as a proof of having found the correct graph structure. On the flip side, we also show that correctly identifying an edge to be activating or inhibiting based on the fitted parameters does not seem to depend excessively on the graph structure used, thus allowing us to deduce with reasonable certainty the relationship between two nodes, if such edge exists in the true graph.

The contributions presented in this work have a place in a broader framework for data-driven modelling. Specifically, our methods find viable configurations of parameters for models of temporal intracellular processes. Based on these parameters, an interesting next step is to identify regions of parameters close to the identified parameters with qualitatively different behaviour. Such an analysis could be conducted through a numerical bifurcation analysis [31 ], a rigorous identification of these parameter regions [18 ] or a combination of both.

We note that our results on parameter fits of an ODE model are dependent on the model equations used. When using another formulation, the model could rely on model parameters specific to that formulation to recover the desired time-series data, which would lead to a different set of alternative hypotheses on the underlying mechanism. Moreover, the loss function used to optimize the model may need to be adjusted if a different model is used. For example, it is often desired that simulations of the model end in an equilibrium state corresponding to the final data point. For some model formulations, this behaviour may not be easy to find, in which case a term could be added to the loss function promoting this behaviour.

Future research could be directed towards different methods for retrieving a dynamic model from data while exploiting the GAE model presented in this article. In particular, SINDy has become a popular method for retrieving nonlinear models based on a library of functions that can be used in the definition of the right-hand side of the ODE [32–34 ]. Thus, a SINDy integration in our code would be our first step in this direction.

We could also consider a more drastic overhaul of our architecture. Indeed, in recent years, neural ODEs have become more prominent in the machine learning community [35 ]. When graph topologies sustain the dynamics, graph neural ODEs ( GDEs ) have also been proposed [36 ]. We foresee two key ways in which neural ODEs can be used to extend the methods presented in this work. First, a GDE could be used in place of the GraphSAGE layers currently in our encoder model. This would allow us to obtain a better view of the global properties of the graph compared with our current architecture, where only information up to a distance of two edges away can be seen. Second, GDEs could be used to directly infer a surrogate model of the dynamical system. If such an approach is successful and provides an accurate (though not easily interpretable) representation of the underlying dynamics, identification of an interpretable model could be done based on fitting the vector field rather than fitting the time series directly. This is a faster method and could lead to improved mechanistic models.

As a direction for future research, it is interesting to consider if adopting a distributional view of the data, using techniques from optimal transport, leads to better results, similar to techniques such as TrajectoryNet and MIOFlow [37,38 ]. Related to these approaches, recent work also shows success in using optimal transport-based approaches to construct stochastic dynamics describing splitting trajectories in single-cell data [39,40 ]. A key question for future work could be whether these approaches can be adjusted to explicitly use an available GRN structure. For example, a graph autoencoder similar to the one used in this work could be used to encode the high-dimensional scRNA-seq data, or a graph neural ODE could be used in place of the multi-layer perceptrons often used in the neural ODE central to these approaches. Such adaptations could improve the interpretability of the models resulting from these methods, and would open the way to the discovery of novel biological hypotheses.

In conclusion, this work presents an application of graph neural networks for constructing mechanistic models for time-dependent gene expression. Our result highlights not only the flexibility of GAEs, but also their limitations, appearing in the form of spurious edges. Furthermore, we demonstrate how a good fit of the data does not relate to a good representation of the underlying model, thus highlighting the importance of additional testing on fitted models, such as the presented knockdown experiments. The novelty of integrating machine learning techniques with additional insights from network dynamics and biology makes strides in showing how GAEs and other methods can be successfully used in practical scenarios to create interpretable models on a case-by-case basis.

## Data Availability

All code used to generate the results and figures shown in this paper can be accessed at GitHub [41] and has been archived with Zenodo [42]. For the generation of synthetic single-cell data, BoolODE was used [43]. We have used six representative graphs provided in this repository for testing our framework: dyn-bifurcating, dyn-bifurcating-converging, dyn-linear, dyn-linear-long, dyn-cycle and dyn-trifurcating. We have used the implementations of GENIE3, PIDC and GRNBOOST2 for benchmarking as included in the BEELINE package [44].
